# Quantitative Magnetic Resonance Cholangiopancreatography Scoring and Its Predictive Value for Outcomes in Adults with Primary Sclerosing Cholangitis

**DOI:** 10.3390/jcm13154548

**Published:** 2024-08-03

**Authors:** Matei Mandea, Speranta Maria Iacob, Mugur Cristian Grasu, Cristian Anghel, Razvan Andrei Iacob, Mihaela Corina Ghioca, Cristian Gheorghe, Liliana Simona Gheorghe

**Affiliations:** 1 Department of Internal Medicine, Discipline of Gastroenterology and Hepatology, University of Medicine and Pharmacy Carol Davila, 050474 Bucharest, Romania; matei.mandea@drd.umfcd.ro (M.M.);; 2 Department of Radiology, Medical Imaging and Interventional Radiology I, University of Medicine and Pharmacy Carol Davila, 050474 Bucharest, Romania; 3 Digestive Diseases and Liver Transplant Center, Fundeni Clinical Institute, 022328 Bucharest, Romania; 4 Laboratory of Radiology and Medical Imaging, Fundeni Clinical Institute, 022328 Bucharest, Romania

**Keywords:** liver fibrosis, primary sclerosing cholangitis, MRCP, prognosis score, liver transplantation, liver-related death

## Abstract

**Background:** Primary sclerosing cholangitis (PSC) is an immune-mediated disease that has an unfavorable prognosis and needs a liver transplant (LT). The aim of this paper was to show the usefulness of the Majoie classification on magnetic resonance cholangiopancreatography (MRCP) images in assessing the prognosis in adult patients with PSC. **Methods:** Our work presents a retrospective monocentric study performed on 64 adult patients with PSC of the large bile ducts. Two radiologists evaluated the MRCP of diagnosis and calculated MRCP scores using the Majoie classification. Liver-related outcome (LT or liver-related death) was marked as a primary endpoint. **Results:** Univariate analysis showed that patients with more severe lesions (sum score of intrahepatic and extrahepatic ducts > 3) had a lower age at diagnosis, of 37.2 years, complicated with liver cirrhosis (53.1% of patients) and recurrent cholangitis (28.1%) *p* < 0.05, without significant differences in mortality, association with IBD or LT. Concordance analysis between MRCP prognostic scores and progression to a PSC-related event showed a moderate relationship (c-statistic 0.662), and a good AUROC was observed for the UKPSC score (0.893) and the MRS (0.936). **Conclusions:** In the study, we observed a good correlation between the imaging scores based on the Majoie classification and the evolution of the patients. These scores were outperformed by the UKPSC, MRS, and PREsTo clinical models. Their utility was best in predicting recurrent cholangitis.

## 1. Introduction

Primary sclerosing cholangitis (PSC) is a chronic inflammatory cholangiopathy defined by irreversible damage to the intra and extrahepatic biliary tree and often associated with inflammatory bowel disease (IBD) [1]. The disease evolves with fibrosis of the bile ducts, leading to cholestasis, bile duct strictures, and hepatic fibrosis with progress towards cirrhosis, increased risk of cholangiocarcinoma, and colorectal cancer [2,3,4,5]. The evolution of the disease is characterized by an essential variability with an overall survival without liver transplant ranging from 13–22 years [5,6].

Another aspect that can influence long-term evolution is the association with other autoimmune diseases, such as autoimmune hepatitis or inflammatory bowel diseases, which must be clarified when a PSC diagnosis is established [4].

The natural history of PSC, with a mean survival of 12–20 years from diagnosis to death or transplant (often longer for asymptomatic patients), has led to a search for prognostic models because of the lack of a validated biomarker. Multiple prognostic scores have emerged, including the widely used revised Mayo risk score (rMRS), Amsterdam–Oxford model (AOM), UK-PSC score, and PSC risk estimate tool (PREsTo). They use noninvasive tests such as total bilirubin, albumin, alkaline phosphatase (ALP), alanine aminotransferase (ALT), platelets, prothrombin time, and clinical features such as age at diagnosis, sex, and small-duct disease [7]. However, these models are primarily used for research and patient stratification, with minimal everyday clinical application. While the Model for End-stage Liver Disease (MELD) and Child–Pugh scores are good options, they have limitations in PSC due to their cholestatic nature [7,8,9,10].

Imaging is crucial in diagnosing PSC through methods like ERCP and MRI. The diagnosis initially performed by ERCP progressed towards using MRCP as the current standard [11]. The use of endoscopic evaluation was gradually abandoned because of the risks that this method carries, such as post-ERCP pancreatitis [12,13]. Imaging prognostic models have been developed to serve as prognostic biomarkers. However, their utility is still being investigated because of limited research. These models aim to assign a quantitative value to imaging features, but a major challenge is the significant variation in interpretations among different readers [10,14].

The Majoie classification was initially described in 1991 for ERCP studies and later validated by Ponsioen et al. [15,16]. in cholangiography studies, assesses strictures in intra- and extrahepatic biliary ducts. It assigns a numerical value to each grade based on lesion severity. Recently, this classification was adapted for use with MRCP images in a pediatric cohort, making it a non-invasive tool. The MRCP score, employing the Majoie classification, showed promise as a prognostic indicator for PSC [17].

The aim of our study was to evaluate the modified Majoie classification in an adult PSC cohort as a prognostic marker and to compare it to other clinical-based models.

## 2. Materials and Methods

Patients diagnosed with primary sclerosing cholangitis (PSC), as determined through magnetic resonance imaging (MRI) assessments, with or without histopathological confirmation via liver biopsy, and corroborated by clinical and paraclinical evaluations, were recruited for this study. The diagnosis was established on typical findings on high-quality MRCP images or liver biopsy after excluding the causes of secondary sclerosing cholangitis and other causes of chronic cholestasis, including Primary Biliary Cholangitis (PBC), based on available guidelines [3,4]. IgG4 status was evaluated only in patients with clinical suspicion of IgG4-related disease. This cohort was identified from the hospital registry and included in the research conducted at our tertiary hepatology and liver transplant center, a single-center institution, from August 2011 to August 2022.

### 2.1. Population of Study

**Figure 1:**
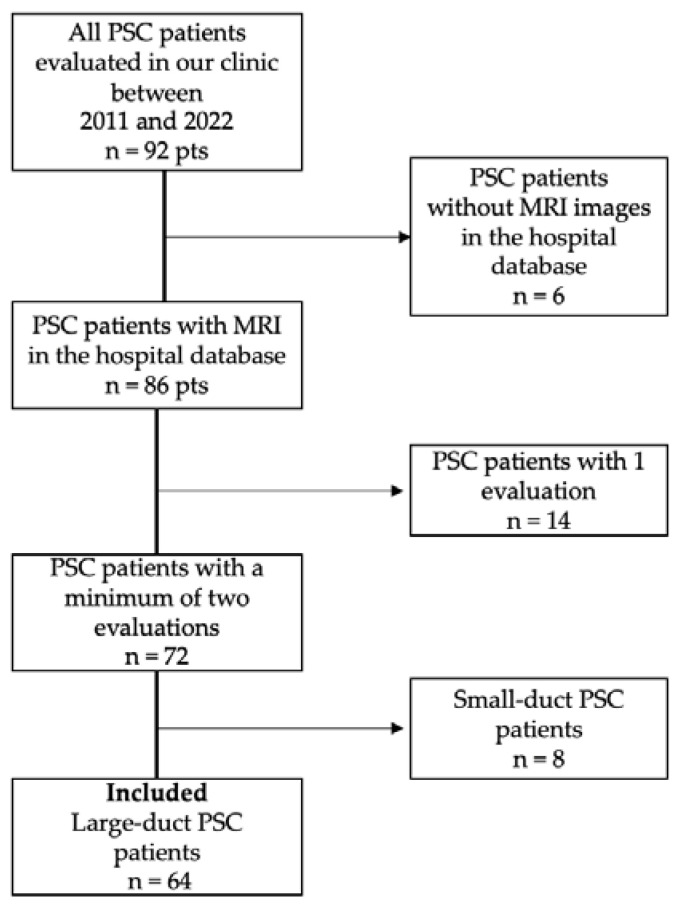
Flowchart presenting the inclusion of patients with diagnosed PSC.

In our study, we included 92 adult patients investigated in our clinic, who were identified by searching the patients from our records using the hospital database. Sixty-four patients had large duct PSC, a homogenous disease feature, with a minimum of 2 evaluations in our clinic, which were included for imaging and prognostic score analysis. Patients who did not have MRI images in the hospital database, who had only one evaluation, and who had small-duct PSC were excluded. The workflow is presented in Figure 1.

For each patient, the clinical information and paraclinical data drawn from the Registry of the Hospital were included. A minimum of one follow-up visit was required for inclusion. The study protocol conformed to the ethical guidelines of the 1975 Declaration of Helsinki and the principles of good clinical practice. All participants provided written informed consent. Ethical approval was granted by the Fundeni Clinical Institute ethical board.

### 2.2. Data Collection and Score Definitions

The imaging studies evaluated in this research were analyzed using the modified version of the Majoie classification, as used by Patil et al. [17] in a pediatric cohort and by Ferrara et al. [18].

The changes observed in the biliary tree during MRCP examinations were categorized according to the classification system used to assess involvement in patients with PSC.

This evaluation was conducted after the radiology team at the Fundeni Clinical Institute analyzed several scores, concluding that this modified imaging scoring of the changes in the bile duct aspect is a type of radiological score that can be easily applied.

This classification uses an adapted form of the original Majoie classification for ERCP [15] and is summarized in Table 1. It uses numerical values assigned for changes identified at the intrahepatic duct (IHD) and extrahepatic bile duct (EHD) levels.

The degree of damage to the bile ducts was noted, and a score of 0 to 3 points was scored for each evaluated segment, as shown in the table.

Intrahepatic bile duct changes were assessed according to the Couinaud anatomical classification, focusing on the ducts within specific anatomical segments.

For the evaluation of the extrahepatic bile ducts (EHDs), the appearance of the common hepatic duct and the appearance of the main bile duct were evaluated.

Two experienced radiologists (M.G., with 25 years of experience, and C.A., with 5 years of experience), aware of the patient’s evaluation indications, visualized and interpreted each MRCP. The latter analyzed the images first, and the former reviewed them.

The included data encompassed age at diagnosis, association with IBD and its specific type, MRCP findings, serological analysis results (including biochemistry, complete blood count, and coagulation profile), and details of any complications that arose during the disease’s progression. Both blood analysis and MRCP data were documented twice. Additionally, the duration of the patient’s follow-up period was recorded.

Several prognostic models, such as the revised Mayo score, the Amsterdam–Oxford model, the UKPSC score, the primary sclerosing cholangitis risk estimate tool (PREsTo), and the Model for End-stage Liver Disease (MELD-Na), were used to assess the outcome of patients with PSC. Scores were calculated for all patients both at the time of inclusion and at the second evaluation performed during the study period or the time of the clinically significant event [19,20].

A clinically significant PSC-related event was defined as liver transplantation (LT) or liver-related death. Recurrent cholangitis was recorded as a separate event.

The types of associated IBD were described according to the endoscopic and histopathological appearances according to standard criteria. Noninvasive scores evaluating liver fibrosis (FIB4 and APRI fibrosis scores) were assessed for each patient.

### 2.3. MRCP Acquisition Technical Data and Protocol

MRI examinations were performed in accordance with the institution’s standardized protocol using one of three available imaging systems. These systems are equipped with phased-array coils and operate at magnetic field strengths of either 1.5 Tesla or 3 Tesla. The institutional protocol for MRCP comprises T1-weighted sequences, heavily T2-weighted sequences, and coronal 3D acquisitions. The distribution of patients to these imaging units was randomized based on the availability of the respective systems.

### 2.4. Statistical Methods

Continuous variables were evaluated using median values and their interquartile ranges (IQRs), whereas categorical variables were assessed based on their occurrence frequencies. The selection of statistical tests, either Fisher’s exact test or the Mann–Whitney U test, was determined by the nature of the analyzed variables.

Concordance analysis for MRCP scores, clinical prognostic scores (AOM, MRS, UKPSC, MELD-Na), and laboratory tests were conducted with Harrell’s C statistical test. The effectiveness of these prognosis scores in predicting PSC-related events was evaluated through ROC (Receiver Operating Characteristic) curve analysis.

Patients were categorized based on their MRCP scores, which were derived from evaluating both intrahepatic and extrahepatic bile ducts. A threshold of 3 was established as the cut-off point for these scores, determined through ROC analysis to optimize sensitivity and specificity for predicting PSC-related events. This threshold allowed for the division of patients into two groups. Further analysis was conducted within these groups to examine the relationship between MRCP scores, clinical prognostic scores, and fibrosis scores, using Spearman rho correlation. This non-parametric test was chosen to measure the strength and direction of the association among these variables, providing insights into the prognostic relevance of MRCP scores in relation to clinical outcomes in PSC patients.

Significance was defined by *p* < 0.05. The analysis was performed using SPSS version 26.0 (IBM Corporation, Armonk, NY, USA), and MedCalc Statistical Software version 22.032 (MedCalc Software bv, Ostend, Belgium; https://www.medcalc.org; 2024)

## 3. Results

**Figure 2:**
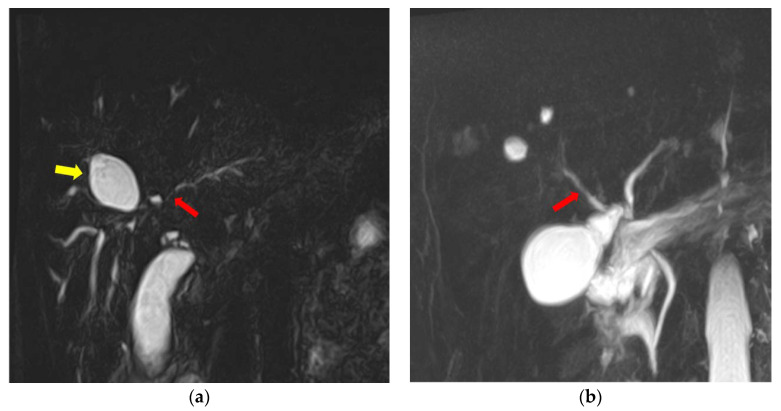
(**a**) MRCP showing multiple segmental strictures affecting both intra- and extrahepatic bile ducts (red arrow), with biliary diverticula present in the right perihilar region (yellow arrow). (**b**) MRCP-MIP reconstruction showing discrete parietal irregularities affecting the intrahepatic bile ducts (red arrow). The images were extracted from Fundeni Clinical Institute records.

Based on the cut-off score of 3 for the SUMIHDEHD, among the 64 patients included in this retrospective study, 49 patients had a score greater than or equal to 3, and 15 patients had a low score (<3). An example of MRCP featuring severe lesions (score SUMIHDEHD = 6) can be seen in Figure 2a, and an example of MRCP with mild lesions (score SUMIHDEHD = 2) is presented in Figure 2b.

The characteristics of the population, the association with IBD, the complications due to PSC, the average period of follow-up, and the results of the clinical prognostic scores are described in Table 2.

The analysis revealed that the relationship between IBD and the severity groups, as determined by the MRCP score-based classification, did not reach statistical significance (*p* = 0.86). On the other hand, the high MRCP score showed statistically significant differences in progression to liver cirrhosis, episodes of acute cholangitis, and time to transplant. Statistically significant differences were also found in relation to the Mayo risk score, MELD-Na scores, the UKPSC short-term prognostic score, and the rate of complications as determined by the PREsTo score at both 1 and 5 years.

The analysis of data on paraclinical changes and fibrosis assessment scores revealed statistically significant disparities between the two patient groups, classified based on their severity as determined by the SUMIHDEHD score, in terms of the APRI fibrosis assessment (average APRI = 1.443, *p* = 0.001) and FIB-4 score (average FIB-4 = 1.70, *p* = 0.023), as well as for total bilirubin (average bilirubin = 2.61, *p* = 0.001). For other liver enzymes indicative of cytolysis and cholestasis, no statistically significant differences were noted between the groups.

Statistically significant differences were observed between the two groups for the average IHD score and for the average AVG SUM IHD-EHD composite score, higher values being observed in the subgroup of patients who presented a PSC-related event in the course of the disease (*p* < 0.05). The presence of acute recurrent bacterial cholangitis indicated a significantly higher average AVG SUM IHD-EHD score in this group compared with patients without recurrent episodes of acute cholangitis or pruritus (2.77 ± 0.73 vs. 2.31 ± 0.81, *p* = 0.01).

### Prediction Analysis

C-statistics were calculated for the follow-up period to predict the progression to a complication due to PSC. In Table 3, the outcomes of Harrell’s C analysis are presented for various MRCP scores as well as for clinical prognostic scores. The imaging scores that showed an increased concordance were the SUM IHD-EHD and IHD scores with a c-statistic of 0.662 and 0.664, respectively. The analysis performed for recurrent cholangitis showed a good concordance with the SUM IHD-EHD score (c-statistic 0.672, SE 0.17, *p* < 0.05).

**Figure 3:**
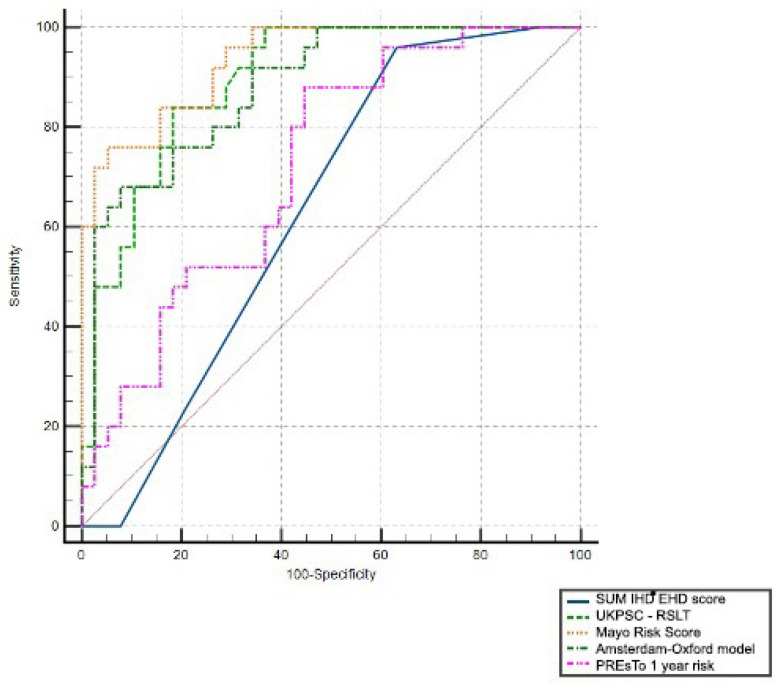
Comparison of ROC curves for PSC prognosis scores.

This study conducted a detailed examination of the capability of clinical and imaging prognostic assessments to differentiate between cases with events related to progressive PSC and those without disease-related complications, utilizing ROC curves. Consequently, a comparison of the ROC curves was carried out, as depicted in Figure 3, including the 1-year PREsTo clinical score, the long-term UKPSC RSLT clinical score, and the MRS and AOM score.

It should be mentioned that the imaging score SUM IHD + EHD showed >95% sensitivity but low specificity (63.2%) for the cut-off score of 3. Clinical scores performed better with a good clinical utility (AUROC > 0.70) to predict a worse outcome, the best being the MRS.

In addition, the MRCP score demonstrated the potential to forecast the necessity for LT in PSC patients, based on a threshold score of 3, achieving a high sensitivity rate of over 90% (93.3%) and a relatively favorable specificity of 71.1%, albeit with an ROC value of 0.63. In comparison, as shown in Table 4, clinical models also presented higher AUROC values, with AOM displaying the most significant result—0.852.

The capacity of the MRCP SUM IHD-EHD score to predict the cases that will present recurrent bacterial cholangitis in evolution was also assessed with an ROC curve, presenting an AUROC of 0.722 with satisfactory sensibility and specificity (94.7% and 68.9%). In this case, the score outperformed every clinical prognosis tool, the most significant result being 0.612 for UKPSC.

The significance of the imaging score was assessed by comparing it against the biological profiles of the patients, including liver enzyme levels and total bilirubin, alongside quantitative clinical prognostic scores and fibrosis evaluation scores (FIB-4 and APRI). A subsequent multivariate Spearman correlation analysis revealed that the two MRCP scores, SUM IHD-EHD and AVG SUM IHD-EHD, showed a notable positive correlation with total bilirubin levels (r = 0.46, *p* < 0.001 for SUM IHD-EHD, and r = 0.47, *p* < 0.001 for AVG SUM IHD-EHD), while correlations with alkaline phosphatase or GGT levels were not significant.

Within the range of clinical prognostic scores, the strongest correlations with the SUM IHD-EHD and AVG SUM IHD-EHD scores were noted for the UK-PSC RSST score (r = 0.48, *p* < 0.001 and r = 0.46, *p* < 0.001, respectively). Additionally, statistically significant positive correlations were also identified with the MRS (r = 0.35, *p* < 0.004 and r = 0.38, *p* < 0.002, respectively). Conversely, the AOM score did not exhibit statistically significant correlations, except for a modest correlation observed solely with the IHD sum score (r = 0.36, *p* < 0.03).

The association between the calculated imaging scores and fibrosis assessments was found to be moderate and statistically significant for the FIB-4 score (r = 0.24, *p* < 0.05 and r = 0.27, *p* < 0.06, respectively). Yet, it was deemed insignificant for the APRI score.

A logistic regression analysis was performed to examine the influence of prognosis scores in predicting PSC-related events. The MRCP score and each clinical score were dichotomized according to the outcome, using ROC curves to obtain cut-offs with optimal sensitivity and specificity. Logistic regression analysis showed that the model as a whole was significant (Chi^2^(5) = 27.38, *p* <0.001, *n* = 64). The MRCP score was the only independent predictor for PSC-related events (*p* = 0.028).

## 4. Discussion

MRCP represents a standard in diagnosing patients with PSC, but it provides qualitative information regarding the severity. The use of a score helps to quantify and stratify the cases to later establish correlations. Our retrospective study evaluated an MRCP score compared to other clinical prognostic scores.

The scoring system used was previously evaluated retrospectively on a pediatric population by Patil et al. [17], who adapted the score initially described by Majoie et al. [15] and adapted by Ponsioen et al. [16]

Comparatively, our study extends this lineage by applying the MRCP-adapted score within an adult Eastern European PSC population, an area not previously explored. This transition from pediatric to adult populations and from ERCP to MRCP underscores the versatility and potential of the scoring system across different patient demographics and diagnostic techniques. Our findings regarding the correlation between MRCP scores and poor prognosis in adult PSC patients offer a new perspective on the score’s predictive validity and clinical utility. Taking into account this context, the present research validates previous data and expands the applicability of the scoring systems to adult patients. This progression signifies an essential step in refining prognostic tools for PSC, suggesting that despite the scoring system’s origins and initial applications, it holds significant relevance for contemporary clinical practice, particularly for predicting the progression of PSC in adults.

In our effort to assess prognosis among the patient cohort, this study incorporated all available scores to date based on the cumulative data for the evaluated patients [21]. Therefore, we calculated the UKPSC score and the PREsTo score, along with the more traditionally employed models in research, namely, the MRS and AOM. The analysis demonstrated high reliability in identifying PSC-related events, with the MRS emerging as the most accurate, evidenced by an AUC of 0.936, and the UK PSC RSLT score as the next most effective, with an AUC of 0.893. These findings underline the utility of these scores in differentiating among varying levels of risk in patients, with particular emphasis on their prognostic value in identifying individuals at higher risk of adverse outcomes. The clinical prognostic scores outperformed the imaging score in predicting LT and liver-related mortality outcomes, demonstrating notably superior outcomes. However, the MRCP score’s value was especially evident in cases where recurrent cholangitis was the outcome. This finding underscores the significance of MRCP imaging in patient assessment and highlights the utility of the SUM IHD-EHD score. As indicated by this score, more severe strictures were identified as a predictive marker for recurrent cholangitis. It is recommended that patients who meet or exceed this score should be promptly referred to a Liver Transplantation Center for further evaluation and should be meticulously monitored in conjunction with the other clinical assessments.

Developed to predict survival in PSC patients, the MRS is a well-established prognostic model that incorporates age, bilirubin, albumin, prothrombin time, and variceal bleeding [22]. Its high accuracy, evidenced by the high AUC in the present study, underscores its effectiveness in identifying patients at increased mortality risk. This score is particularly valued for its robust validation across different cohorts and its ability to guide clinical decision-making, especially when considering LT.

The UKPSC score was designed to assess prognosis in patients with PSC, reflecting various clinical, biochemical, and histological parameters to predict outcomes such as LT, complications, or mortality related to the disease. The UK-PSC score outperformed the revised MRS (rMRS) in both the derivation (c-statistic 0.81 for R_ST_ vs. 0.75 for rMRS and 0.80 for R_LT_ vs. 0.79 for rMRS) and validation cohorts (c-statistic 0.81 for R_ST_ vs. 0.73 for rMRS and 0.85 for R_LT_ vs. 0.69 for rMRS) [23], different from our findings. The PREsTo score is a newer addition to the landscape of PSC prognostic tools. Designed to predict the risk of PSC-related complications, such as cholangiocarcinoma, LT, or death, PREsTo uses a combination of clinical and biochemical parameters. In the initial validation cohort, PREsTo compared favorably to the rMRS (c-statistic 0.85), MELD score (c-statistic 0.85), and AP < 1.5 ULN (c-statistic 0.65) [21]. However, our results showed a totally different result with the lowest prognostic value for the PREsTo score compared with the other clinical scores.

The predictive role of the two fibrosis scores, FIB-4 and APRI, was not significant in the study population, and weak correlations were observed with severity in MRCP scores, these results having a weak significance.

The data observed in the analysis of imaging scores are comparable to those previously identified due to the distribution pattern of peribiliary fibrosis in PSC, but this cannot be assessed using the FIB-4 and APRI clinical scores to determine the degree of liver fibrosis [10,24].

In future research, MRI elastography could significantly enhance patient monitoring by providing a comprehensive assessment during the same exploratory session. This technique, which measures liver stiffness as an indicator of fibrosis, has shown promising results in studies [24,25] and could offer a valuable comparison or complement to traditional fibrosis scoring systems such as Fib-4 and APRI. Incorporating MRI elastography could yield a more nuanced understanding of PSC severity and progression, potentially improving patient outcomes through more informed clinical decision-making.

Considering the need to quantify the radiological changes observed in MRCP in PSC patients, several classification systems and prognostic scores have been developed. One is the ANALI score, developed by Ruiz, Lemoinne et al. [26]. This simple score evaluates MRI images with and without gadolinium contrast administration. This score was externally validated and has good correlations with survival without liver transplantation and decompensated liver cirrhosis, with c-statistic values of 0.89 and 0.75 [26,27].

Recently, considering the technological advances in the development of artificial intelligence and its applicability in the field of radiology, a software program that analyzes MRCP images in patients with PSC was developed (MRCP+ software, https://www.perspectum.com/our-products/mrcpplus-rollback-content-21apr23, Perspectum Diagnostics Limited, Oxford, UK), evaluated for the prediction of events related to PSC, in comparison with other assessment methods such as MRE or clinical scores. This application was used to automatically evaluate the images and results presented by Selvaraj et al. and Ismail et al. [28,29]. The results showed significant correlations with the other clinical scores and the ANALI score, pointing out the patients at high risk of developing complications [30].

This system has been externally validated but also combined with other parameters, such as spleen size, number of strictures, total bilirubin, and the aspartate aminotransferase level, to develop scores with an improved prognostic ability for PSC-related complications and mortality due to PSC [31,32].

Another prognostic tool, the DiStrict score, developed by Grigoriadis et al. [33], leverages 3D MRCP imagery to quantify disease severity through numerical ratings of bile duct lesions, both intrahepatic and extrahepatic. This approach mirrors the scoring methodology applied in the current study but exhibits improved alignment with liver-related outcomes. This enhancement in concordance may stem from the score’s direct derivation from MRCP images. Essentially, both scores aim to quantify the severity of bile duct strictures and the occurrence of dilations, underscoring their role in assessing disease progression.

Recurrent cholangitis represents a substantial complication in PSC management, given its potential to exacerbate liver damage, accelerate disease progression, and significantly impair patient quality of life. The direct link between a high MRCP score, indicative of more extensive bile duct involvement, and a higher rate of recurrent cholangitis highlights a critical aspect of disease burden that is not directly accounted for by the MELD score. Exception points for patients with recurrent cholangitis and/or pruritus due to PSC, in addition to the current MELD allocation system, acknowledge the additional disease burden and risks these patients face [34].

Overall, the literature indicates that while MRCP and clinical prognostic scores each have their strengths, combining them may offer a more nuanced and accurate approach to predicting disease progression and outcomes in PSC patients. This integrated approach can help tailor patient management strategies and improve prognosis by identifying high-risk patients who may benefit from closer monitoring or early intervention.

Despite its retrospective nature, the present study encompasses several strengths that underscore its contributions to understanding PSC progression. Firstly, it features a substantial cohort of patients, each followed meticulously until the endpoint of decompensation of liver cirrhosis, death, or liver transplantation, ensuring a comprehensive analysis of disease progression. Secondly, this study notes a lower association with IBD compared with what is commonly reported in the literature, offering new insights into the disease’s epidemiology [1]. Our finding also suggests that the severity of the liver condition, as measured by MRCP, may not be directly influenced by the co-occurrence of IBD in patients with PSC. This score, compared with other widely recognized and validated clinical prognostic scores, enhances our ability to predict PSC outcomes and identify patients with liver cirrhosis and those at higher risk of adverse progression. The extended duration of patient monitoring further adds to this study’s robustness, providing valuable longitudinal data on PSC evolution and prognosis.

The MRCP score demonstrated strong correlations with other clinical prognostic indicators and the progression towards more severe outcomes in adults with PSC, particularly highlighting the significance of intrahepatic bile duct lesion analysis. However, the score did not perform better than clinical prognosis models in predicting combined PSC-related outcomes, its importance being the most relevant in indicating recurrent cholangitis. This score can be readily applied by radiologists examining PSC patient images, and its clinical relevance should be further explored in a prospective study featuring systematically gathered data.

This study has several limitations, primarily due to its retrospective design. Also, an assessment of the status regarding autoimmunity markers present in these patients, such as serum perinuclear Anti-Neutrophil Cytoplasm Antibodies (pANCAs), Anti-Saccharomyces cerevisiae (ASCA), or IgG4 status, was not uniformly performed, so the exclusion of these data from the information collected and analyzed represents another limitation. The presence of pANCAs in patients with PSC was previously identified in 70–80%. However, this antibody is not disease-specific and has limited implications for patients’ clinical aspects and evolution.

The fact that different MRI devices were used to evaluate the patients, both 1.5 T and 3.0 T, in addition to the evaluation of the images by non-blinded radiologists from the diagnosis of the patients and without performing an inter-rater analysis, represents the limitations of the present study. In essence, our work builds on the foundation laid by previous studies, providing valuable insights into the MRCP scoring system’s effectiveness in an adult population. This enriches the existing literature and offers a basis for future research to explore further and optimize prognostic scoring systems for PSC, enhancing patient care and management strategies. In addition, it highlights the necessity of integrating both imaging and clinical data, including the consideration of exception points, to ensure a comprehensive and equitable approach to LT prioritization in PSC patients where the MELD score does not reflect the full impact of PSC on a patient’s health and prognosis.

## 5. Conclusions

In conclusion, the score adapted according to the Majoie classification was validated on a cohort of adult patients. It shows a good predictive ability for the evolution of the patients and for the occurrence of liver-related outcomes, such as mortality and the need to perform LT. This score was compared with other clinical scores that showed stronger correlations with the outcomes. Prospective comparative studies with other imaging scores are needed to establish the definite role of this score in clinical practice.

## Figures and Tables

**Table 1:** Score definitions for the modified Majoie classification calculated on MRCP (from Ferrara et al., Majoie et al., Rajaram et al., and Patil et al. [14,15,17,18]).

Score	Definition
IHD 0	No abnormalities
IHD 1	Minimum stenosis with biliary ducts of regular diameter or minimally dilated
IHD 2	Multiple stenosis and saccular dilations with reduced intraparenchymal arborization
IHD 3	Closed stenosis to carrefour with obstruction or lack of visualization of one of the main hepatic ducts
EHD 0	No abnormalities
EHD 1	Wall irregularity without significant stenosis
EHD 2	Segmental stenosis
EHD 3	Entire stenosis of CBD
IHD score (total)	Worst score of the IHD analysis score
EHD score (total)	Worst score of the EHD analysis score
SUM IHD-EHD score	Sum of the IHD score (total) + EHD score (total)
Average IHD score	The average between all IHD segments, calculated by anatomical classification
Average EHD score	The average between all IHD segments calculated by anatomical classification
Average SUM IHD-EHD score	Sum of the average IHD and average EHD scores

IHD, intrahepatic duct; EHD, extrahepatic duct; CBD, common bile duct.

**Table 2:** Population characteristics.

Variable N (%) or Average (IQR)	All PSC (N = 64)	SUM IHD-EHD ≥ 3 (N = 49)	SUM IHD-EHD < 3 (N = 15)	*p* Value
Female	35 (54%)	26 (40.6%)	13 (20.3%)	0.630
Age at diagnosis (years)	43.8 (15–69)	37.29 (23–49)	47 (38.5–57)	0.029
Follow-up (months)	46 (1–120)	43.6	53.6	0.270
Time MRI-OLT (months)	35.6	30	114	0.105
IBD	16 (25%)	12 (18.7%)	4 (6.2%)	0.866
UC	9 (14%)	7 (10.9%)	2 (3.1%)	
CD	7 (11%)	5 (7.8%)	2 (3.1%)	
Liver cirrhosis	36 (56%)	34 (53.1%)	2 (3.1%)	0.0001
Death	13 (20.3%)	13 (20.3%)	0	0.060
OLT	15 (23.4%)	14 (21.8%)	1	0.082
Neoplasm	5 (7.8%)	5 (7.8%)	0	0.201
Acute recurrent cholangitis episodes	19 (29.6%)	18 (28.1%)	1	0.001
MRS inclusion	0.426 (−0.329–1.201)	0.649 (0.012–1.212)	0.289 (−1.045–0.110)	0.003
MRS follow-up	0.850 (−0.390–1.201)	1.262 (0.055–2.692)	−0.467 (−1.015–0.170)	0.0001
AOM inclusion	2.011 (1.455–2.648)	2.073 (1.452–2.692)	1.815 (1.465–2.130)	0.287
AOM follow-up	2.236 (1.525–2.946)	2.453 (1.452–2.722)	1.542 (1.185–1.800)	0.002
MELD-Na inclusion	10.8 (7–14)	11.7 (8–15)	8.06 (7–9.5)	0.002
MELD-Na follow-up	14 (7–16)	15.8 (8–18.5)	8.2 (7–9)	0.002
UKPSC RSST	−2.574 (−3.324–−1.736)	−2.365 (−3.165–−1.536)	−3.242 (−3.540–−3.172)	0.002
UKPSC RSLT	−1.184 (−2.231–0.310)	−0.921 (−1.863–0.086)	−2.024 (−2.792–−1.656)	0.004
PREsTo 1 year	4.91%	5.99%	1.43%	0.002
PREsTo 5 years	20.64%	24.41%	8.61%	0.002

IQR, interquartile range; PSC, primary sclerosing cholangitis; IHD, intrahepatic duct; EHD, extrahepatic duct; MRI, magnetic resonance imaging; OLT, orthotopic liver transplant; IBD, inflammatory bowel disease; UC, ulcerative colitis; CD, Crohn’s disease; MRS, Mayo risk score; AOM, Amsterdam–Oxford model; MELD, Model for End-stage Liver Disease; RSST, short-term risk score; RSLT, long-term risk score; PREsTo, primary sclerosing cholangitis risk estimate tool.

**Table 3:** Harrell’s C statistical analysis for concordance between MRCP and clinical prognosis scores and progression to a PSC-related event.

Score	C-Statistic for PSC-Related Event (SE)
MRCP IHD score	0.664 (0.36)
MRCP EHD score	0.542 (0.43)
MRCP SUM IHD-EHD score	0.662 (0.20)
MRCP average IHD score	0.586 (0.53)
MRCP average EHD score	0.551 (0.54)
MRS follow-up score	0.539 (0.15)
AOM follow-up score	0.556 (0.24)
MELD-Na score	0.613 (0.05)
UKPSC RSST	0.471 (0.20)
UKPSC RSLT	0.545 (0.19)

Numbers are presented as value; PSC, primary sclerosing cholangitis; SE, standard error; MRCP, magnetic resonance cholangiopancreatography; IHD, intrahepatic duct; EHD, extrahepatic duct; MRS, Mayo risk score; AOM, Amsterdam–Oxford model; MELD, Model for End-stage Liver Disease; RSST, short-term risk score; RSLT, long-term risk score.

**Table 4:** Calculated AUC for PSC prognosis scores.

Variable	AUC	SE	95% CI	*p* Value
SUM IHD-EHD score	0.631	0.0678	0.500 to 0.749	0.004
PREsTo 1-year risk score	0.721	0.0640	0.594 to 0.827	<0.001
UKPSC long-term risk score	0.893	0.0388	0.790 to 0.957	<0.001
MRS score	0.936	0.0280	0.844 to 0.982	<0.001
AOM score	0.880	0.0424	0.773 to 0.948	<0.001

Numbers are presented as value; AUC, area under ROC curve; SE, standard error; CI, confidence interval; IHD, intrahepatic duct; EHD, extrahepatic duct; PREsTo, primary sclerosing cholangitis risk estimate tool; MRS, Mayo risk score; AOM, Amsterdam–Oxford model.

## Data Availability

The data on which this study is based will be made available upon request.
